# Adolescents’ Behaviors, Fitness, and Knowledge Related to Active Living before and during the COVID-19 Pandemic: A Repeated Cross-Sectional Analysis

**DOI:** 10.3390/ijerph19052560

**Published:** 2022-02-23

**Authors:** Senlin Chen, Baofu Wang, Stacy Imagbe, Xiangli Gu, Jared Androzzi, Yang Liu, Sami R. Yli-Piipari, Gang Hu, Amanda E. Staiano

**Affiliations:** 1 School of Kinesiology, Louisiana State University, Baton Rouge, LA 70803, USA; bwang34@lsu.edu (B.W.); simagb1@lsu.edu (S.I.); 2 Department of Kinesiology, University of Texas at Arlington, Arlington, TX 76019, USA; xiangli.gu@uta.edu; 3 Department of Physical Education, Sport and Human Performance, Winthrop University, Rock Hill, SC 29733, USA; androzzij@winthrop.edu; 4 Department of Physical Education, Wuhan University of Technology, Wuhan 430070, China; worldinsight@163.com; 5 Department of Kinesiology, University of Georgia, Athens, GA 30602, USA; syp@uga.edu; 6 Pennington Biomedical Research Center, Baton Rouge, LA 70808, USA; gang.hu@pbrc.edu (G.H.); amanda.staiano@pbrc.edu (A.E.S.)

**Keywords:** COVID-19, fitness education, middle school, physical activity, sedentary behavior

## Abstract

Background: Nearly all schools in the United States experienced shutdown followed by phased reopening during the COVID-19 pandemic, thereby limiting students’ opportunities for physical activity (PA). This study aimed to examine adolescents’ PA at school (PAS) and PA out-of-school (PAO), screen-based sedentary behaviors (SbSB), health-related fitness, and knowledge understanding about PA and fitness before and during the pandemic. Methods: Three rounds of data were collected: Time 1 pre-pandemic (January 2020; *n* = 405), Time 2 schools partially reopened (February 2021; *n* = 412), and Time 3 schools fully reopened (March 2021; *n* = 450). Adolescents completed the Youth Activity Profile, the 20 m Progressive Aerobic Cardiovascular Endurance Run (PACER) test and Plank test, and a written test, to measure the behaviors (T1, T2, T3), fitness (T2–T3), and knowledge (T1, T2, T3), respectively. Results: Inferential statistical analyses revealed a significant time effect for the behaviors and fitness. From T1 to T2 PAO decreased but PAS increased; whereas SbSB decreased at T3 compared to T1 and T2. Health-related fitness improved from T2 to T3. Further, the change patterns for SbSB varied by grade, and those for knowledge understanding varied by gender. Conclusion: The findings confirm the disruptive impact of the COVID-19 pandemic on adolescents’ active living but varied by school grade and gender. The favorable changes from T2 to T3 observed for fitness and other constructs may be partially attributable to an interrupted fitness education intervention. The findings may guide the design and evaluation of future interventions addressing the physical inactivity pandemic during public health crises (e.g., COVID-19).

## 1. Introduction

School is an important setting for children and adolescents to accrue movement time needed to meet the physical activity (PA) guidelines [1]. Alleviation of long-standing upward trajectories of youth physical inactivity are ensured with consistent attendance at schools which provide safe spaces to be active [2]. PA within the context of school settings increases youth socialization opportunities to lead active lives [3]. Opportunities for PA may stem from school-based programs including physical education (PE) classes, recess, classrooms, before and after school programs, as well as out-of-school sources such as youth sports, home activities, and play [4,5]. Equally important, youth sport participation is tied to meaningful physical health contexts such as increased PA levels, improved competence (e.g., physical literacy), and psychosocial outcomes [6]. Active living is associated with numerous health benefits [1] and can be achieved through regular participation in health-enhancing PAs, maintaining a modest level of physical fitness, and engaging in reduced and frequently interrupted sedentary behavior (i.e., limiting screen-based sedentary behavior (SbSB) in particular) [7]. Many interventions, both in and beyond the school setting, have been implemented to address the physical inactivity pandemic, however, a vast majority of U.S. adolescents (12–19 years old) have failed to meet the PA guidelines [1] or other related guidelines (e.g., the 24 h movement guidelines) [8,9]. Recently, the COVID-19 pandemic and its related policy restrictions have had a long-lasting impact on adolescents’ daily living worldwide [10], with a major influence that millions of schools were forced to halt in-person instruction for virtual or hybrid instruction [11]. While these alternative models of instruction (along with effective vaccines and other public health measures) have contributed to containment of the virus, millions of school-aged youth also experienced reduced access to PA opportunities both in and out of schools, thereby increasing their exposure to screen time, hampering PA participation, and exacerbating challenges to well-being [12,13,14]. This study aimed to investigate adolescents’ behaviors, fitness, and knowledge related to active living before the pandemic and during two phases of school re-opening.

Since early spring 2020, the global pandemic of COVID-19 has brought up varying degrees of impact to factors related to active living. These impacts (large or small) could be direct or indirect but varied across different countries and geographical locations with different countering strategies. Although the direct impact of the SARS-CoV-2 virus on the health of the healthy, younger population (e.g., hospitalization rate, mortality rate) has been significantly less than that of the unhealthy and/or older populations [10], the pandemic incurred policy, social, and environmental changes, including but not limited to government lockdowns, stay at home orders, limited accessibility to parks and recreational facilities, mask wearing, social distancing, or perceived threat, have collectively resulted in substantial, indirect impact on their health-related behaviors and health outcomes [15,16,17]. For example, one large-scale national survey in the USA found that children and adolescents’ PA level, especially for moderate-to-vigorous PA and community-based peer PA participation, significantly declined during the pandemic, as compared to pre-pandemic [18]. A recent systematic review of eight studies found that the lockdowns during the pandemic significantly reduced children and adolescents’ frequency of PA participation and disturbed their sleep [19]. Another scoping review of 84 empirical studies further reported the decline in daily PA ranging from 10.8 to 91.0 min [20].

The global pandemic might have affected adolescents’ behaviors, fitness, and learning related to active living to a different extent across sociodemographic groups, which warrants empirical research [21]. Of the factors, in this study we examined the change patterns by gender, grade in school, and race. Disparities in PA participation, health, and education have existed both before and during the COVID-19 pandemic. Prior to the pandemic, boys, girls, and other gender types showed different levels of PA (and SbSB) in terms of interest, choice, amount, or intensity [22,23]. Boys were often reported to be more active yet also more sedentary than girls [24]. They preferred vigorous intensity and competitive PA, while girls preferred recreational, rhythmic, and cooperative, PAs with lower to moderate intensity [25,26]. Adolescents of sexual or gender minorities (e.g., lesbian, gay, bisexual, transgender, queer, and questioning; LGBTQ+) were found to be a marginalized, at-risk population who perceive PA insecurity and inconvenience and therefore do not participate in PA regularly [27]. One recent scoping review of the literature found that the decrease in PA during the COVID-19 pandemic was more pronounced in boys compared to other gender groups [28], but it is not clear if these gender differences are specific to PA during in- vs. out-of-school time or if these differences extend to fitness and knowledge related to active living.

School grade level or age is another demographic factor of consideration, when it comes to health and education equity. Prior to the pandemic, large-scale surveillance data indicated that PA levels steadily declined from childhood to adolescence [21]. During middle school years, eighth grade students were often less physically active and engaged in higher levels of SbSB. One recent study found that the disruptive impact of COVID-19 on youth PA varied in terms of amount, type, and intensity, but its impact was greater on high school students than that on preschool students [18]. Another study reported that during the early phase of COVID-19 pandemic, youth aged 9–13 showed greater decline in PA behavior than their younger counterparts (5–8 years old) [29]. The potential impact of school closures and re-opening across early adolescence is less documented. Furthermore, aside from school grade level or age, race is also an important potential indicator of health inequity. Racial or ethnic minorities such as Black or Hispanics were previously reported to display less favorable PA behaviors and fitness conditions, and lower levels of knowledge understanding related to active living [7]. The COVID-19 pandemic might have exacerbated the health disparities between different racial and ethnic populations and social classes. In fact, some epidemiologic evidence has demonstrated a greater, direct impact of the COVID-19 pandemic on the economically disadvantaged racial minorities (e.g., rates of hospitality and mortality), compared to their White counterparts, especially during early and mid-phases of the pandemic [10].

As synthesized above, the COVID-19 pandemic has had a significant impact on some, if not all, constructs related to active living. Our literature review has identified several gaps that can be filled through this current study. First, many studies have examined adolescents’ PA and SbSB in light of the COVID-19 pandemic, but little research has focused on fitness and knowledge related to active living. Second, existing studies have largely examined the pandemic’s disruptive impact using a pre-to-post design or the one-shot observational design. Rarely have they compared its disruptive impact on active living during different phased reopening periods. Third, little research has longitudinally examined the disruptive impact across demographic groups during the phased reopening periods. Therefore, the primary research purpose was to examine the changes in active living behaviors (i.e., PA at and out of school, SbSB), health-related fitness (i.e., cardiorespiratory endurance, abdominal endurance), and knowledge related to active living before and during two reopening phases of the pandemic across three time points. The secondary research purpose was to discern the changing patterns of these outcome variables by gender, grade in school, and race.

## 2. Materials and Methods

### 2.1. Research Design

This study followed the repeated cross-sectional research design, which is a type of longitudinal research design [30]. The data of this study were collected at three time points, from adolescents enrolled in a public middle school located in the southern Louisiana, USA. The school and researchers maintained a partnership since the spring of 2018 collaborating in a series of projects. As part of the partnership, the researchers provided the teachers with professional development (e.g., assessment of PA and health-related physical fitness, sharing curricular and instructional strategies and resources). In return, the students and teachers contributed data to inform and address meaningful research questions. The lessons learned from these collaborative efforts had been jointly presented to peer PE teachers and other practitioners from schools across the state at an annual conference for PE teachers. In the spring semester of 2020, in particular, we attempted to conduct a high-intensity interval training (HIIT) curriculum intervention experiment at the school where classes at each grade level were randomized to the experimental or comparison groups. Baseline assessments (T1) of students’ active living behaviors and knowledge were administered to both groups of classes in mid-January, followed by implementation of an externally designed fitness education curriculum unit. However, the implementation was halted in February, as the COVID-19 pandemic forced the school to shut down its in-person instruction. As a result, the school adopted virtual instruction for the reminder of the spring semester. Between fall 2020 and mid-February 2021 (T2), the school transitioned from virtual to hybrid instruction, with gradually more students returning to school for in-person instruction (partial reopening). For hybrid instruction attendees, students were split into halves, alternating between in-person versus virtual attendance days. A proportion of students attended virtually throughout the semesters, although that percentage declined as perceived pandemic risk dwindled over time. From late January 2021, the PE teachers decided to teach the HIIT fitness education lessons to all students. Since mid-February 2021, when T2 assessments were conducted, the school fully resumed in-person instruction. T3 assessments were administered in late March 2021, upon the completion of implementing all 14 HIIT-based fitness education lessons. The study protocol was approved by the Louisiana State University Institutional Review Board and the participating school’s principal. Informed parental consent and assent were completed prior to data collection.

### 2.2. Setting and Participants

The participating school is a public, Title I, suburban middle school. According to the database of National Center for Education Statistics (http://nces.ed.gov (accessed on 29 October 2021)), in the 2019–2020 academic year (i.e., the most recent available data), the school enrolled 496 students in 6th (*n* = 180), 7th (*n* = 149), and 8th (*n* = 167) grades with a 19:1 student teacher ratio, roughly even number of boys (*n* = 244) and girls (*n* = 252), mostly White (*n* = 256) and Black (*n* = 206) students, and more than half of the students eligible for free (*n* = 259; 52.2%) or reduced-price (*n* = 29; 1.8%) lunch. At the time of data collection (January 2020), the PE and health education program was taught by two experienced, certified specialists who had been teaching at the school for over eight years. The PE program at the school recently changed from daily 50 min instruction combined for 6th, 7th, and 8th grades to every-other-day 50 min instruction separated by grade. This change resulted in 50% reduction in PE instructional time as well as substantial decrease in class sizes and student/teacher ratios. In January to February 2020, the PE program had half of the students receiving the HIIT fitness education, and the other half receiving regular multi-activity based PE; and then the experiment was discontinued due to the pandemic disruption. Between January and March 2021, the teachers implemented the HIIT fitness education lessons in all classes. As stated earlier, the school experienced phased reopening with growing students attending regular, in-person instruction later in the spring.

The adolescent participants were in 6th, 7th, and 8th grades at each time of data collection. Because the data collection cycle spanned two academic years, the 8th grade students in their first year advanced to high school (9th grade) and a new cohort of 6th grade students joined the middle school in the second year. The original 6th and 7th grade students in the first year remained in the study, but became 7th and 8th grade students, respectively, in their second year. The participants completed the Youth Activity Profile, the 20 m Progressive Aerobic Cardiovascular Endurance Run (PACER) test and the Plank test, and a validated written test, to measure behaviors (T1–T3), fitness (T2–T3), and knowledge (T1–T3), respectively. The sample sizes varied across the three measurement time points (*n =* 405, January 2020; *n =* 412, February 2021; *n =* 450, March 2021). Table 1 shows the sample sizes by gender, grade, race, and time of measurement.

### 2.3. Variables and Instrumentation

#### 2.3.1. Behaviors

Adolescents’ active living behaviors including PA at school (PAS), PA out-of-school (PAO), and SbSB were measured using a previously validated self-report tool called Youth Activity Profile (YAP; Saint-Maurice and Welk, 2015). The instrument has 15 questions capturing the above three behaviors (5 items per behavior). One example question that asks about PAS is phrased as: How much physical activity did you do last Saturday? (this could be for exercise, work/chore, family outings, sports, dance, or place. If you cannot remember, try to estimate). Possible answers were: A. No activity (0 min), B. Small amount of activity (1–30 min), C. Small to moderate amount of activity (31–60 min), D. Moderate to large amount of activity (1–2 h), E. Large amount of activity (more than 2 h). The validity and reliability of the YAP have been previously examined against valid objective measures [31], and it has been previously used in similar school-based research [32].

#### 2.3.2. Health-Related Fitness

Adolescents’ health-related fitness, cardiorespiratory endurance, and abdominal strength in particular, was assessed using the validated FitnessGram 20 m Progressive Aerobic Cardiovascular Endurance Run (PACER) [33] and the Plank Assessment of Torso Strength [34]. The 20 m PACER was administered by following the standard FitnessGram Protocol on the school’s court space of the multi-purpose gymnasium [35]. The adolescents, in groups of 8–12, upon familiarity and practice, ran as many laps as possible in straight lines back and forth following the pre-determined signals and music. Their number of completed laps were recorded to capture cardiorespiratory endurance. Similarly, administration of the Plank test followed the standard testing instructions [34]. The participants took the test in groups of 15–20 and reported their time (in seconds) to the recording teacher. Correct form of the test (e.g., feet together, toes under feet, perfectly straight body line) was required and reinforced to maximize assessment accuracy. Both the PACER and the Plank tests had been previously used before in the PE program, so both the teachers and students were familiar with them.

#### 2.3.3. Knowledge

Adolescents’ knowledge understanding pertinent to active living was measured using a written test. The 22-item test encompassed 15 validated items adopted from a PE Metrics middle school knowledge test [36], three validated items from the FitSmart test [37], and 4 new items developed by the researchers. These 22 items were purposely selected or developed to thoroughly assess the content scope of the HIIT fitness education curriculum unit, which was implemented in the curriculum intervention experiment in spring 2020 (before the pandemic) and then again in spring 2021 (phased reopening). The four new items established adequate content and construct validity (Imagbe et al., in press). All items had one single correct answer. Responses to the questions were scored following the answer key. The number of correct responses were tallied and subsequently averaged by the number of questions, to determine the level of knowledge understanding (% correct responses). One item that tested knowledge about the FITT principle is phrased as: The FITT principle of training has four components. They are Frequency, Intensity, Type and          . Possible choices were: a. Task, b. Time (correct answer), c. Tempo, d. Target.

### 2.4. Data Collection

Data collection occurred at three time points (T1: January 2020; T2: February 2021; and T3: March 2021). At all three time points, participants’ behaviors and knowledge of active living were assessed using the same online Qualtrics survey that included the YAP, knowledge test, and several demographic questions (i.e., grade, gender, class). Staff from the school’s media center transported the laptop computers (with connected WiFi) to the gymnasium during the designated PE lessons for the participants to complete the survey. Each student completed the survey on Qualtrics on a school-provided laptop computer during the first part of each PE lesson (10–15 min), and then the PE teachers engaged the participants in regular PE activities for the remainder of the lessons. On a different day, with one week apart from the survey data collection time at T2 and T3, the participants also completed the PACER and Plank tests. Two PE teachers administered the tests with research staff support. These two teachers had previously received standardized training and had relevant experience of giving the tests. As instructed in the PACER test manual, participants were asked to stop the test after running a maximum of 75 laps. Each class completed the PACER test within 30 min. The Plank test was administered shortly after the PACER test, which was completed within 10 min. A trained researcher visited the school at T1 and T3, to monitor the data collection processes and provide support when needed. The data collection protocols were adhered to and, therefore, completed efficiently as expected. Make-up tests were arranged for participants who were absent or excused from testing during the regular data collection days.

### 2.5. Data Processing and Analysis

Data collected from the three time points were processed and organized retrospectively between May and December of 2021. Online survey data were downloaded from the Qualtrics server and saved as an Excel file to the hard drive of a data processing computer. The responses to the YAP items were subsequently aggregated by the three constructs (PAS, PAO, SbSB). All but three items were anchored at the 5-point scale (3 items on the 6-point scale). Fitness data were manually entered into the same Excel spreadsheet. All data collected from different sources (online survey, fitness data) and all measurement time points were merged into one single Excel spreadsheet, which was subsequently converted to a SPSS file for analyses. Multivariate analysis of variance (MANOVA), repeated measures MANOVA, and ANOVA were conducted to examine the overall time (T1, T2, T3) effect for the three behaviors combined, two fitness scores (PACER and Plank test results), and knowledge, respectively. Subsequently, these three sets of inferential statistical analyses tested the interactions of time with gender (i.e., boys vs. girls; other genders excluded from inferential analysis due to small sample size), grade (6th, 7th, or 8th grades), and then race (Black/African Americans, White/Caucasian, Other). The univariate tests of between-subjects effects were automatically generated following each of the above multivariate analyses. Significance level (*α*) was set at 0.05 and partial eta square ($\eta_{p}^{2})$. values were reported as effect sizes.

## 3. Results

**Figure 1:**
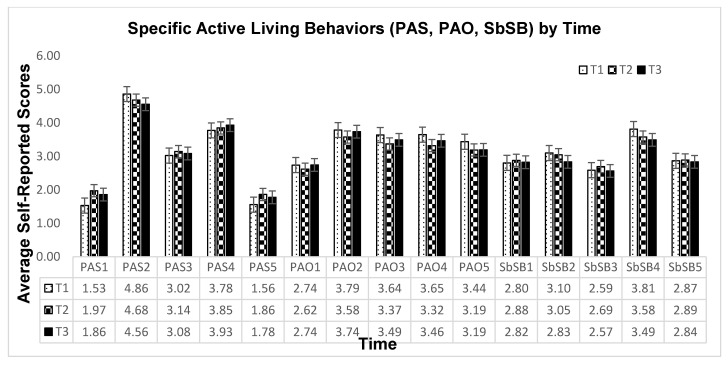
The average levels of specific active living behaviors at each time point. PAS: physical activity at school; PAO: physical activity out of school; SbSB: screen-based sedentary behavior. PAS1–PAS5: walk/bike to school, PE, breaks/study hall, lunch break, walk/bike from school; PAO1–PAO5: before school, after school, school day evening, Saturday, Sunday; SbSB1–5: viewing TV, playing video games, using computers, using cellphones, sitting habits. T1 = January 2020; T2 = February 2021; T3 = March 2021.

To address the primary research purpose, we retrospectively examined the participants’ behaviors (T1, T2, T3), fitness (T2, T3), and knowledge (T1, T2, T3) from a longitudinal perspective. Table 2 shows the descriptive results for these variables at each measurement point. The MANOVA with three behaviors (PAS, PAO, SbSB) as the outcome variables showed a significant time effect (Box’s M = 20.93, *p* > 0.05; Wilk’s Lambda = 0.97, *F* = 6.34, *p* < 0.05). The subsequent tests of between-subjects effects showed significant time effect for all three behaviors (PAS: *F*_2,1153_ = 4.02, *p* < 0.05, $\eta_{p}^{2}=0.007;$. PAO: *F*_2,1153_ = 5.98, *p* < 0.01, $\eta_{p}^{2}$ = 0.010; SbSB: *F*_2,1153_ = 3.09, $\eta_{p}^{2}=0.005$). As shown in Table 2, PAO significantly decreased from T1 to T2 (*p* < 0.01) while PAS significantly increased (*p* < 0.05). SbSB was significantly lower in T3 compared to T1 and T2 (*p* < 0.05). To further illustrate the levels of the active living behaviors across settings by time, we plotted Figure 1 using the raw scores based on the responses to individual items of the YAP. For health-related fitness, repeated measures MANOVA revealed a significant time effect (*p* < 0.01, $\eta_{p}^{2}=0.22$). Specifically, the follow-up tests of between-subjects effects found significant increases of PACER laps completed (*F* = 71.71, *p* < 0.01; $\eta_{p}^{2}=0.182$) and Plank time (*F* = 18.22, *p* < 0.01; $\eta_{p}^{2}=0.056$) from T2 to T3. In addition, knowledge understanding about active living did not show a statistically significant time effect (*p* > 0.05).

**Figure 2:**
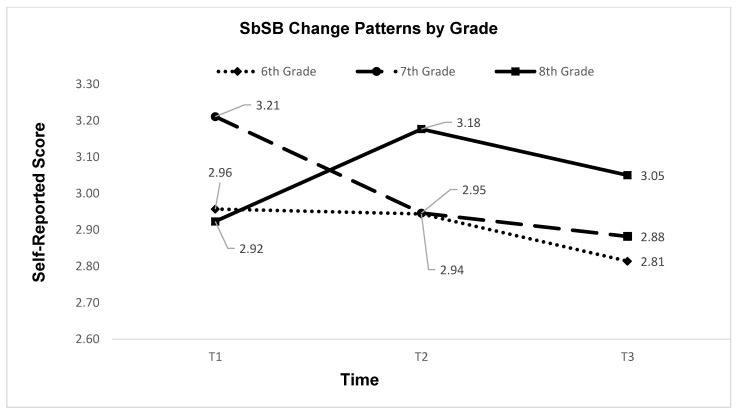
Screen-based sedentary behaviors (SbSB) by time and grade. T1 = January 2020; T2 = February 2021; T3 = March 2021.

**Figure 3:**
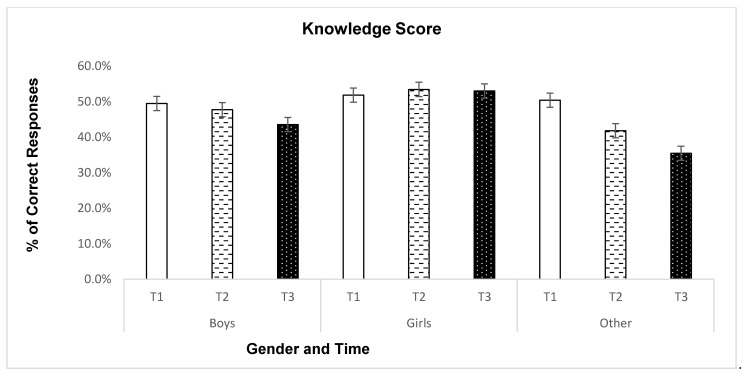
Knowledge understanding about active living by gender and time. T1 = January 2020; T2 = February 2021; T3 = March 2021. Other gender: LGBTQ+ or students who did not report gender.

To address the secondary research purpose, we examined the longitudinal changes of the three sets of outcomes, separately, by gender, grade, and race. The MANOVA with active living behaviors as outcome variables showed a significant time*grade interaction effect (Box’s *M* = 57.51, *p* > 0.05; Wilk’s Lambda = 0.98, *F* = 2.00, *p* < 0.05, $\eta_{p}^{2}=0.007$), whereas the interactions of time with gender or race were not statistically significant. Subsequent univariate analysis located the significant interaction effect to be with SbSB (*F* = 4.15, *p* < 0.01, $\eta_{p}^{2}=0.014$). Table 3 shows the descriptive results for the three active living behaviors by time and grade. Furthermore, Figure 2 illustrates the specific change patterns of SbSB by grade and time. In addition, health-related fitness results did not show any interaction effects (*p* > 0.05). Last but not the least, knowledge understanding about active living demonstrated a statistically significant time*gender interaction effect (*F*_2,1239_ = 4.78, $\eta_{p}^{2}=0.008$), but no other significant interactions of time with grade or race. Figure 3 illustrates the time*gender interaction for the knowledge.

## 4. Discussion

The results of this study addressed two research purposes: (a) to examine the changes of the behaviors, fitness, and knowledge related to active living before and during two reopening phases of the COVID-19 pandemic; and (b) to examine the changing patterns of these outcome variables by gender, grade, and race. Based on repeated cross-sectional analyses, our results unraveled the varying, disruptive impact of the COVID-19 pandemic on adolescents’ active living constructs by time and demographic characteristics (i.e., grade and gender). These findings are discussed below and may shed light to the design and evaluation of future interventions to address the physical inactivity pandemic.

One significant disruptive impact of the COVID-19 pandemic is the observed decline in PAO from T1 (before pandemic) and T2 (partial reopening). This decline, as illustrated in Figure 1, appears to be consistent on weekdays (i.e., before school, after school, and evening hours) and weekend days (Saturday and Sunday). Decreased PA in light of the pandemic has been repeatedly reported in many prior studies [19,20]. During the lockdown as well as the subsequent phased reopening time periods, adolescents’ PA behaviors have been largely restricted by dramatic societal changes ranging from limited open hours of parks and recreational facilities, modified instructional models and schedules in schools, and parents working from home (with increased parental distress and home chaos), to the new norms of social distancing and mask mandates [38]. The perceived threat of the pandemic, though varying across groups and individuals over time, remained modest to high, which hampered individuals’ PA participation. Both structured and unstructured out-of-school opportunities of PA such as youth sports programs and play were curtailed, to various extents, as one of the counter strategies for mitigating community spread of the virus.

Our repeated cross-sectional analysis also found an unexpected increase in PAS from T1 to T2. At T2, the school had the vast majority of students attending in-person instruction altogether, many of whom recently transitioned from a rotation schedule (with one half students in-person and the other half virtual on one day; then rotate on the next day). Based on our interactions with the teachers, the school did not add new opportunities of PAS at T2. However, upon a further look at the specific PAS responses, as illustrated in Figure 1, the participants reported reduced PA during PE but increased PA through active transportation to and from school, during class breaks/study hall, and lunch break. The reduced PAS during PE makes sense as the school indeed shortened its PE duration from 45 to 30 min per lesson during its initial reopening phase (i.e., T2). The higher levels of PAS through active transportation, class breaks, and recess are novel findings. These findings suggest that on school days during the reopening, students chose to walk or bike more frequently to/from school and that teachers encouraged their students to move around more within class breaks and lunch recess, especially outside of the school building (e.g., track, playground, parking lot). The students might have also preferred to be active, as inside their classrooms were often stricter COVID-19-related policies (e.g., indoor mask mandate).

Another set of inspiring findings of the study are that SbSB decreased at T3 from T1 or T2 (no difference between T1 and T2) and health-related fitness improved from T2 to T3. The changing patterns of SbSB should be further analyzed by grade level, as our results also showed significant time*grade interaction. Although we did not observe significant overall change from T1 to T2, the trajectories were quite different by grade. Specifically, as illustrated in Figure 2, 8th grade participants increased their SbSB, 7th grade participants decreased their SbSB, while 6th grade participants did not show any visible change. The increase in SbSB was expected, as previous studies reported higher accessibility of electronic devices during the pandemic. Our finding suggests that rather than examining global changes in SbSB, analyses of screen time should take into account differences by adolescents’ age or grade level. Further, the decrease in SbSB from T2 to T3 appears consistent for the participants of all three grade levels. The results shown in Figure 1 indicate that the decreasing trend of SbSB, especially from T2 to T3, was universal for TV viewing, playing computer/video games, using computers, using cellphones, as well as for the overall sitting habits. The reopening has brought some normalcy to the adolescents, including the return of after-school activities such as sports. A return to regular bedtimes may also contribute to reduced screen time. In addition, the improvement in health-related fitness (i.e., cardiorespiratory endurance and abdominal strength) was another encouraging result. As stated in Section 2, the school was implementing a fitness education curriculum designed by the researchers. The initial version of the curriculum demonstrated preliminary efficacy in improving students’ learning, PA, and fitness [32]. Therefore, this enhanced version of the curriculum might have contributed to the participants’ fitness, although we were unable to assess a control group in this repeated cross-sectional study.

Last but not the least, we did not observe a significant main time effect for knowledge understanding related to active living. The participants, overall, did not change their knowledge understanding in light of the pandemic. However, our subsequent analysis detected a significant time*gender interaction effect. As illustrated in Figure 3, girls showed overall higher knowledge understanding than boys and other genders. More importantly, girls’ knowledge understanding maintained across the three time points, while boys and other genders (especially the latter) showed a decreasing trend. The results favoring girls are consistent with prior research, which usually found girls outperforming boys in health-related knowledge [39]. However, rarely has prior research examined gender differences during the COVID-19 pandemic by also adding other genders to the analysis. Teaching adolescents to acquire a solid understanding about active living is an essential correlate of lifelong PA participation. Future research and education should particularly address the gender gap in learning such knowledge through purposeful physical and health education.

To our knowledge, this is one of the few longitudinal studies that have simultaneously investigated the disruptive impact of the COVID-19 pandemic on adolescents’ behaviors, fitness, and knowledge related to active living across demographic groups (i.e., gender, grade, and race). Despite the novel findings, we acknowledge the following limitations. First, this study collected data from one public middle school in a southern USA state. The observed trends as well as other findings may be generalizable only to 6th, 7th, and 8th grade students enrolled in schools with similar characteristics. However, we were successful in recruiting and retaining most of the students in all three rounds of data collection, in light of the pandemic. A related limitation is that we were unable to track the retention or attrition of participants at the student level in this repeated cross-sectional study. For example, 8th grade students at T1 went onto 9th grade at T2 and T3, 6th grade students at T2 or T3 were still in 5th grade, and 7th grade students at T1 became 8th graders. We only retained de-identified data, which limited our ability to match and track the participants at the individual student level. A longitudinal analysis with intact data from individual students would potentially reflect a more accurate data analysis, but that would also significantly reduce sample size. The other limitation is that the active living behaviors were measured using a self-report measure. This measure, however, has previously demonstrated sound validity and feasibility in assessing PAS, PAO, and SbSB in middle school students. We also used valid tests to measure cardiorespiratory and muscular fitness, as well as knowledge understanding related to active living. Further, active living constructs (e.g., behaviors, fitness, knowledge) may be influenced by a number of environmental or sociocultural factors (e.g., socioeconomic status), which were not taken into account in this study. Future research may assess these factors and take them into account for an ecological analysis.

These study findings bear significant implications to research, practice, and policy. With the constant mutations of the virus (e.g., Omicron variant), the COVID-19 pandemic and its disruptive impact are expected to linger, to a greater or lesser extent, in the foreseeable future. Future research should continue to monitor students’ levels of behaviors, fitness, and knowledge related to active living, to identify facilitators/barriers and put forth theory-guided, tailored interventions, which would ultimately minimize the disruptive impact of the pandemic on these constructs for all students (e.g., all genders, races/ethnicities, grade levels). In practice, most schools have resumed in-person instruction, with caution. Resuming in-person instruction in schools as well as after-school programs or youth sports programs have normalized the offering of PA opportunities and therefore the participation levels of PA. These normalcies are expected to reverse students’ declining trend for PAO and increasing trend for SbSB. Furthermore, regular school-based instructions such as physical and health education are needed, as shown in the results (e.g., improved fitness from T2 to T3), to improve and sustain their health-related physical fitness and potentially other learning outcomes. As far as policy implication is concerned, with the waning disruptive impact of the pandemic in recent months, policy makers in schools (e.g., principals, school board), communities (e.g., park and recreational departments), and home (e.g., parents) should work in tandem to maximize students’ opportunities to adopt and maintain active lifestyles. The findings from this study may inform future active living interventions to effectively target factors on multiple socioecological levels (e.g., policy and environmental, community, organizational, social, and interpersonal factors) across settings both in and out of school, to ultimately increase PA and fitness levels, curb SbSB, and also improve academic outcomes.

## 5. Conclusions

The COVID-19 pandemic has brought up dramatic societal changes worldwide. Understanding the disruptive impact of the pandemic on constructs related to active living is a crucial topic of interest. Based on repeated cross-sectional analyses, this study unraveled adolescents’ change patterns of behaviors, fitness, and knowledge related to active living across three demographic groups before and during the pandemic. The findings confirm the pandemic’s varying disruptive impact on adolescents’ active living constructs by grade and gender. The favorable changes from T2 to T3 observed for health-related fitness and other constructs by school grade or gender may be attributable to the HIIT fitness education intervention. However, due to the pandemic, the experimental research design was modified (lost the control group). Therefore, these speculated findings need to be confirmed through a future, controlled study. In conclusion, the pandemic has limited some adolescents’ opportunities to be active, fit, and to learn. Tailored interventions both in and beyond the school setting are needed to address and minimize these disruptive impacts from public health crises such as the COVID-19 pandemic.

## Figures and Tables

**Table 1:** Demographic characteristics of the sample across three time points.

Variables	Subgroups	T1 (Jan 2020)	T2 (Feb 2021)	T3 (Mar 2021)
Gender	Boys	191	187	212
	Girls	203	220	232
	Other	11	5	5
Grade	6th	165	147	152
	7th	137	141	168
	8th	103	124	129
Race	Black	157	142	155
	White	166	178	182
	Other races	82	92	113

Note. T1 = January 2020; T2 *=* February 2021; T3 = March 2021. Other gender = LGBTQ+ or students who did not report gender.

**Table 2:** Descriptive results for the behaviors, fitness, and knowledge by time—M (SD).

Time	PAS	PAO	SbSB	PACER	Plank	Knowledge
T1	2.95 (0.70) *	3.45 (0.98) *	3.03 (0.79) ^a^	-	-	50.7% (13.9%)
T2	3.10 (0.76) *	3.22 (0.91) *	3.02 (0.76) ^b^	12.21 (6.04)	72.54 (30.09)	50.7% (15.3%)
T3	3.04 (0.78)	3.33 (0.91)	2.91 (0.72) ^ab^	15.03 (7.86)	78.77 (27.69)	48.3% (20.3%)

Note. T1 = January 2020; T2 *=* February 2021; T3 = March 2021. PAS: physical activity at school; PAO: physical activity out of school; SbSB: screen-based sedentary behavior; PACER: Progressive Aerobic Cardiovascular Endurance Run. * *p* < 0.05. ^a^ denotes significant difference between T1 and T3 for SbSB; ^b^ denotes significant difference be-tween T2 and T3 for SbSB.

**Table 3:** Descriptive results for the behaviors by time and grade—M (SD).

Grade	Time	PAS	PAO	SbSB
6th Grade	T1	3.05 (0.68)	3.68 (0.98)	2.96 (0.81)
	T2	3.25 (0.79)	3.27 (0.86)	2.94 (0.75)
	T3	3.16 (0.74)	3.48 (0.83)	2.81 (0.76)
7th Grade	T1	2.97 (0.68)	3.27 (0.89)	3.21 (0.74)
	T2	3.07 (0.67)	3.25 (0.97)	2.95 (0.77)
	T3	3.05 (0.78)	3.28 (0.97)	2.88 (0.69)
8th Grade	T1	2.78 (0.72)	3.32 (1.01)	2.92 (0.79)
	T2	2.97 (0.79)	3.12 (0.90)	3.18 (0.75)
	T3	2.90 (0.82)	3.21 (0.90)	3.05 (0.69)

Note. T1 = January 2020; T2 *=* February 2021; T3 = March 2021. PAS: physical activity at school; PAO: physical activity out of school; SbSB: screen-based sedentary behavior.

## Data Availability

The raw data supporting the conclusions of this article may be made available by the authors, without undue reservation, upon written request.
